# Cryopreservation method for spheroids and fabrication of scaffold-free tubular constructs

**DOI:** 10.1371/journal.pone.0230428

**Published:** 2020-04-02

**Authors:** Kenichi Arai, Daiki Murata, Shoko Takao, Ana Raquel Verissiomo, Koichi Nakayama

**Affiliations:** Department of Regenerative Medicine and Biomedical Engineering, Faculty of Medicine, Saga University, Saga, Japan; Michigan Technological University, UNITED STATES

## Abstract

Cryopreservation is a method used for preserving living cells by cooling them to very low temperatures. Although cryopreservation methods for oocytes and embryos have been developed for use in reproductive medicine, there are no established methods yet for preserving cell aggregates (spheroids) in regenerative medicine. We have developed a bio-three-dimensional (3D) printer that can fabricate scaffold-free 3D constructs by loading spheroids onto a needle array. We fabricated several constructs such as blood vessels, liver, diaphragm, and a conduit for nerves by using this method. These constructs have the potential to be applied in patients. However, the process of fabricating tissue constructs (harvesting cells, expanding cells, making spheroids using cultured cells, printing constructs, and maturing constructs) is time-consuming. Therefore, cryopreservation methods for spheroids or constructs should be developed to increase the efficiency of this method for clinical use. Here, we developed a method for cryopreserving spheroids, which were then used to fabricate constructs. Fibroblast cell-based spheroids were cryopreserved in phosphate-buffered saline or cryopreservation solution at −80°C for 1 week. After thawing, spheroids in cryopreservation solution began to fuse on day 1. Cryopreserved spheroids were printed onto a needle array to fabricate a scaffold-free tubular construct using a bio-3D printer. After 7 days, the printed spheroids fused and formed scaffold-free constructs. We confirmed the viability of cells in the cryopreserved spheroids and fabricated tubular constructs. Our results indicate that spheroids can be cryopreserved and used to prepare scaffold-free constructs for clinical use.

## 1. Introduction

Living cells obtained from tissues can be cryopreserved for an extended period in cryopreservation solution [1–3]. Several research groups have improved the cryopreservation solution and method for cryopreserving human induced pluripotent stem (iPS) cells and other cell types [4–8]. Cryopreservation methods can be classified into two types: slow-freezing and vitrification methods [9]. The differences between these technologies are because of varying the concentrations of cryopreservation solutions and rates of cooling. In the slow-freezing method, the cryopreservation solution contains cryoprotectants such as dimethyl sulfoxide (Me_2_SO), which prevents ice crystal formation that leads to cell damage and death. Nearly all somatic cell lines are cryopreserved using this method. Currently, cell viability and the undifferentiated state of human iPS cells can be maintained using the slow-freezing method. The cells can also be cryopreserved effectively using the vitrification method. Vitrification methods have been used for oocytes and embryos in the field of reproductive medicine [10–13]. However, the cryopreserved cells must be thawed as quickly as possible to prevent recrystallization because the viabilities of cryopreserved cells decrease if they are thawed slowly [9]. The cryopreservation solution volume and cooling rate also affect crystallization during freezing and recrystallization during thawing [14]. Thus, it is challenging to cryopreserve large tissues such as cell aggregates (spheroids) and micro-tissues.

A previous study of the cryopreservation of hepatocyte spheroids [15] showed that a large number of cells (78%) in spheroids survived after thawing by using the University of Wisconsin solution containing 15% Me_2_SO. This cryopreservation solution has excellent potential for application to other cell types in spheroids, but this has not been evaluated. Moreover, trials for cryopreserving testicular tissues are underway, but this technique requires further development [12,13]. Cryopreservation technology must be optimized in terms of the cryopreservation solution and protocol for each cell type in spheroids.

Cryopreservation technologies for spheroids are useful in regenerative medicine and tissue engineering. The long period of spheroid preservation can be applied not only in alternative therapy for organ transplantation but also in the development of new drugs. In a previous study, we developed a bio-three-dimensional (3D) printer that can load spheroids onto a needle array according to a desired 3D design. Our system uses a needle array rather than a scaffold, and thus, the fabricated construct is scaffold-free. By using this bio-3D printer, several tissues such as blood vessels, liver, diaphragm, and cardiac tissues and conduits for nerve regeneration were fabricated [16–20]. These fabricated tissues and organs can be applied clinically in the near future. However, the process for fabricating tissue constructs (harvesting cells, expanding cells, making spheroids using cultured cells, printing constructs, and maturing constructs) is time-consuming.

**Fig 1:**
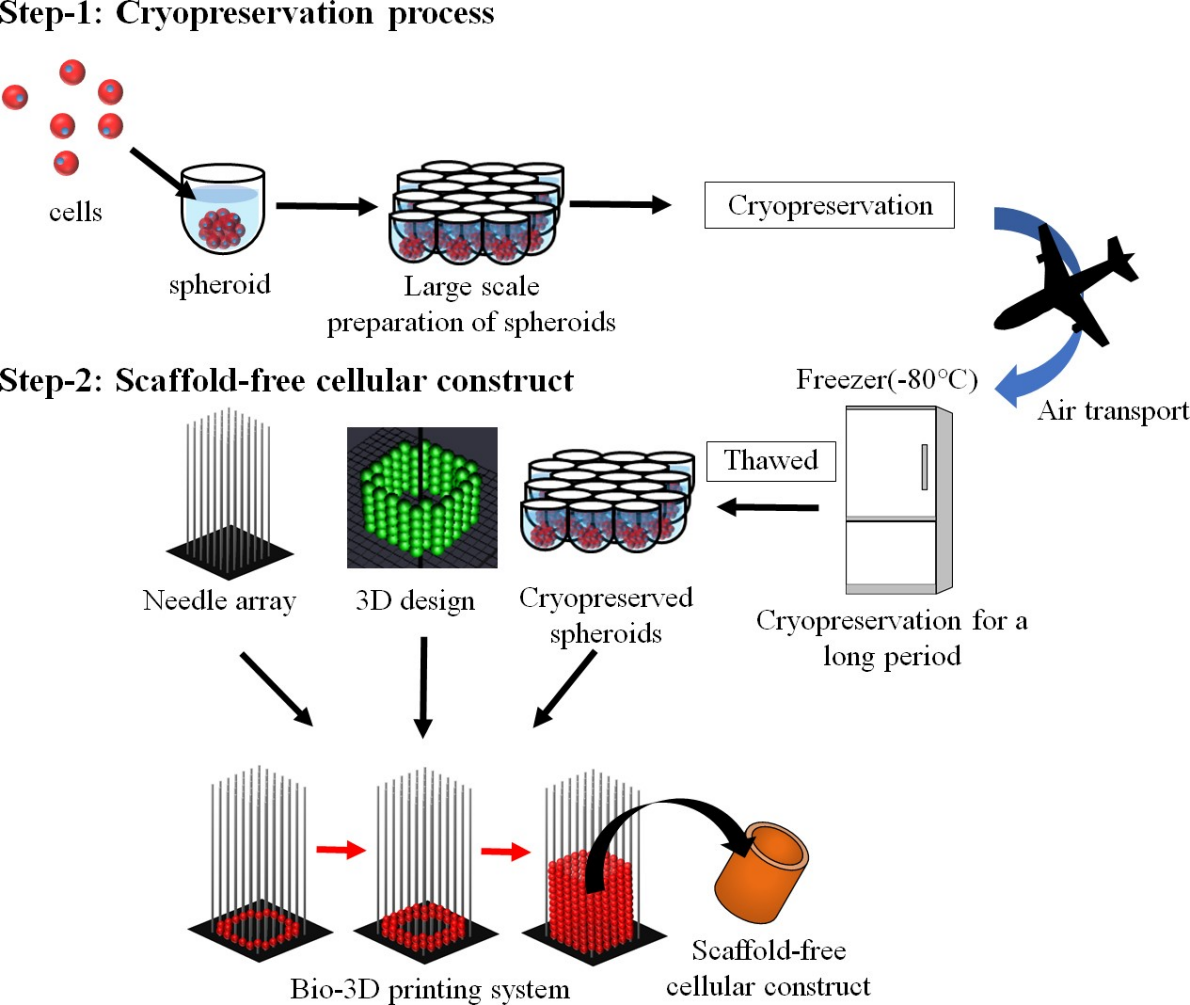
Scheme of the research strategy. Step 1 shows the cryopreservation process of normal human dermal fibroblast (NHDF) spheroids. Spheroids are prepared in a 96-well plate and frozen. These spheroids can be stored for a long period until clinical application. Step 2 shows the bio-3D printing system. After the spheroids are thawed, they are set up on the bio-3D printer, together with the needle array and 3D design. The spheroids are loaded onto the needle array according to the 3D design. Next, the spheroids are fused for a few days after loading.

Thus, engineered tissue constructs cannot be prepared immediately, even when patients must be treated as soon as possible. To overcome this problem, a cryopreservation system for spheroids will be useful for preparing scaffold-free constructs in a short time (Fig 1).

We previously used fibroblast-based spheroids to fabricate cellular constructs by using a bio-3D printer [17,19,20]. This Kenzan method for spheroid-based 3D bioprinting is useful for clinical applications, and we developed a cryopreservation technique for fibroblast-based spheroids. We first prepared spheroids and cryopreserved them at −80°C for 1 week by using the slow-freezing method. We then examined the fusion and cell viability of the cryopreserved spheroids. Moreover, we fabricated tubular constructs using cryopreserved spheroids and confirmed spheroid fusion on a needle array.

## 2. Materials and methods

### 2.1. Cell culture

Normal human dermal fibroblasts (NHDFs) were purchased from Lonza (Basel, Switzerland) and cultured in fibroblast growth medium in a humidified atmosphere at 37°C and 5% CO_2_ in an incubator. The cells were passaged every few days until the required number of cells was obtained.

### 2.2. Spheroid formation

NHDFs were seeded into ultra-low attachment 96-well plates (Primesurface^®^, Sumitomo Bakelite, Tokyo, Japan); 2 days later, the cells had formed spheroids, each containing 30,000 cells, and the spheroids were then cryopreserved. As a non-cryopreserved positive control, spheroids containing 25,000 cells were formed and used to fabricate cellular tubular constructs. These spheroids were cultured in a humidified 37°C and 5% CO_2_ incubator and then used in the experiments. The size and roundness of spheroids were measured by bio-3D printer software. The formula used to calculate roundness of spheroids was as follows:

$$Circle\;rate(\%)=\frac{100-(R-r)}{100R}$$

where “R” is the radius of the minimum circumscribed circle, and “r” is the inscribed circle’s radius.

### 2.3. Cryopreservation techniques

First, these spheroids were collected into cryotubes (Thermo Fisher Scientific, Waltham, MA, USA) and washed with phosphate-buffered saline (PBS). Next, the spheroids were soaked in 200 μL of cryopreservation solution (StemSure^®^; Wako Pure Chemical Industries, Osaka, Japan) or PBS (as a negative control) for cryopreservation. These cryotubes were placed in a bio-freezing vessel (Bicell^®^; Nihon Freezer Co., Ltd., Tokyo, Japan), transferred to a −80°C freezer, and stored for 7 days.

To thaw the spheroids, 1 mL of warmed cell culture medium was added to the cryotubes. These spheroids were mixed gently by pipetting for thawing, and the supernatant medium was removed from the cryotubes. Next, 1 mL of medium was added to the cryotubes, and the spheroids were washed with cell culture medium using a microtube mixer (Intelli-Mixer; ELMI Ltd., Aizkraukle, Latvia). These washing processes were repeated 0, 1, 3, and 5 times.

### 2.4. Fusion activity of cryopreserved spheroids

After thawing, three cryopreserved spheroids were placed in a well and cultured. Spheroid fusion was determined by digital microscopy (MC120 HD; Leica, Wetzlar, Germany). As a positive control, non-cryopreserved spheroids were used.

### 2.5. Fabrication of cellular tubular constructs by using a bio-3D printer

**Fig 2:**
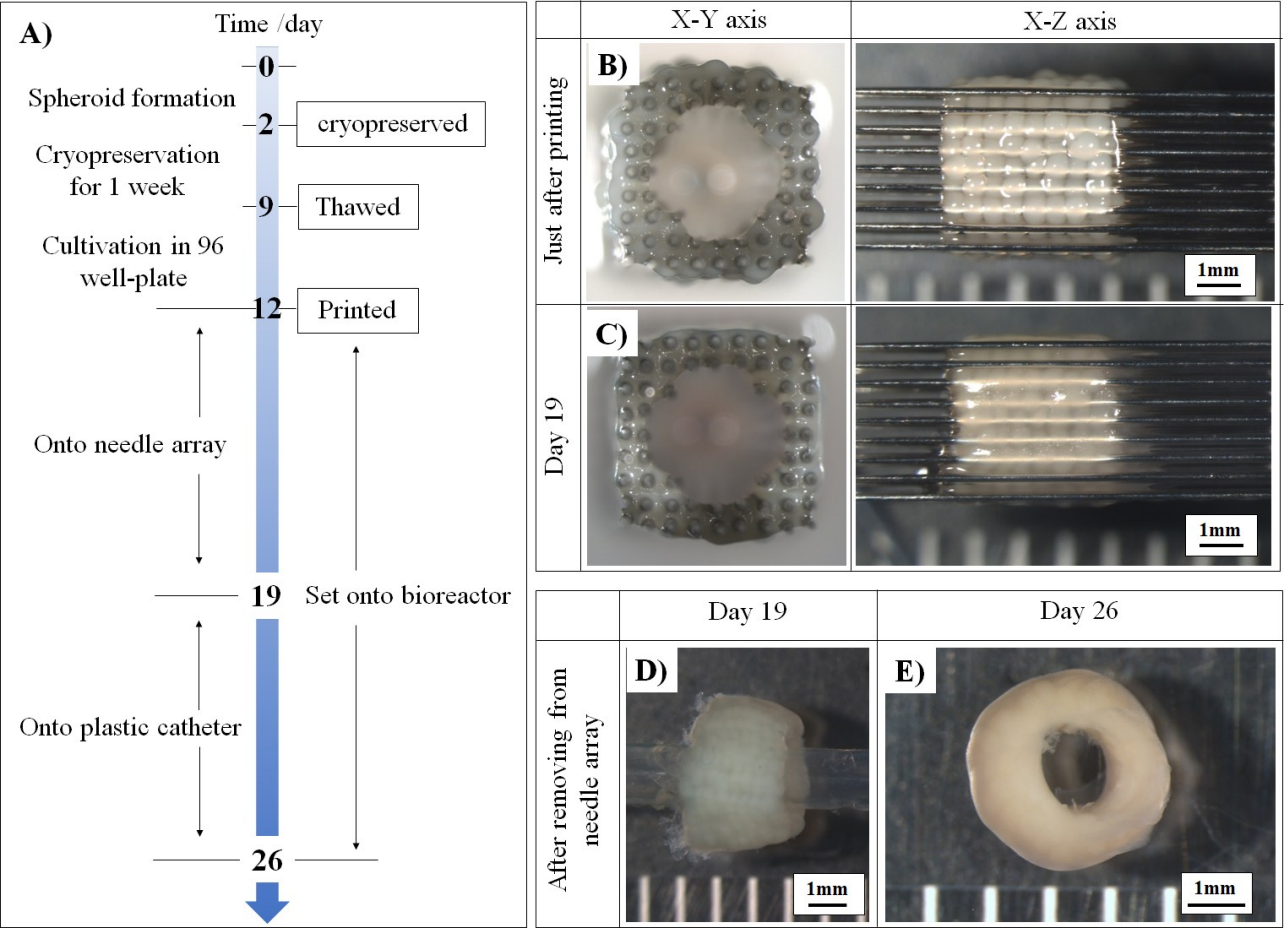
Fabrication of the scaffold-free tubular construct using cryopreserved spheroids. Bio-3D printing process using cryopreserved spheroids (A). The fabricated tubular construct just after printing (B) was cultured on the needle array for 7 days (C). The cryopreserved tubular construct was set on the plastic catheter after removing the construct from the needle array (D), and maturation occurred for 7 days after culture (E). Scale bar, 1mm in (B, C, D, E).

Thawed spheroids were cultured for 3 days, and their size and roundness were evaluated with Cell Accumu software (Cyfuse Biomedical K.K., Tokyo, Japan) in a bio-3D printer (Regenova®; Cyfuse Biomedical K.K.). The bio-3D printer was used to attach the spheroids to the needle array to fabricate scaffold-free tubular constructs, as previously reported [16–20]. Fig 2A shows the fabrication process of the constructs using cryopreserved spheroids. These cryopreserved and thawed spheroids were printed onto the needle array by using a bio-3D printer (Fig 2B). These spheroids in the fabricated tubular constructs were confirmed to fuse with each other after 7 days (Fig 2C). The tubular constructs were removed from the needle array and cultured on a plastic catheter for 7 days (Fig 2D and 2E).

### 2.6. Histological analysis

Spheroids and constructs were fixed in 10% neutral-buffered formalin (Wako Pure Chemical Industries, Ltd.) for 24 h at 4°C. After fixation, the samples were washed three times with PBS, embedded in paraffin, and cut into 5 μm sections. These sections were stained with hematoxylin and eosin or Masson’s trichrome stain and used for immunostaining. Primary antibodies against collagen type Ⅰ (dilution 1:1000; ab34710, Abcam Plc., Cambridge, UK) was used. We used an *in situ* cell death detection kit (Roche Applied Science, Basel, Switzerland) according to the manufacturer’s instructions to confirm cell viability in the TUNEL-stained sections. Stained slides were analyzed using a microscope (BZ-X700; Keyence, Osaka, Japan). TUNEL-positive stained cells in the pictures were counted by using ImageJ software (National Institutes of Health, Bethesda, MD, USA).

### 2.7. Cell proliferation assay

First, the cryopreserved spheroids were collected into 50 ml centrifuge tubes (Corning, NY, USA) and washed with PBS. Next, the spheroids were soaked in 1 mL of cell dissociation solution (Accumax, Innovative Cell Technologies, CA, USA) and incubated at 37°C for 20 min. Next, the spheroids were gently pipetted up and down until a single-cell suspension was obtained. The single-cell suspension was then seeded in a dish, and the number of cells on the 1^st^ and 3^rd^ days of culture was measured using cellular ATP measurement (CellTiter-Glo 3D cell viability assay, Promega, Madison, WI, USA). As a positive control, non-cryopreserved spheroids were used.

### 2.8. Tensile strength test of constructs

The tensile strength of the constructs using cryopreserved spheroids was measured with a tensometer (Tissue Puller-560TP; Bio Research Center, Aichi, Japan), according to the manufacturer’s instructions. The tensile strength of each construct was measured to determine uniaxial tension. The force at failure of each construct was calculated using MyoPULL software (Bio Research Center, Aichi, Japan), as the maximum load that the construct could withstand. We measured the length, thickness, and diameter of the constructs. The constructs were subjected to cyclic tension loading until failure. The stress (mN/mm^2^) of the construct was plotted with MyoPULL software, creating a stress-strain curve. These constructs were cultured for 14, 21, and 28 days after fabrication and measured.

### 2.9. Statistical analysis

Data are expressed as means ± SD. The values represent the means ± SD from three independent experiments. Comparisons between the two groups were analyzed using a Student’s t-test. A P value < 0.05 indicates statistically significant differences.

## 3. Results

### 3.1. Fusion of cryopreserved and thawed spheroids

**Fig 3:**
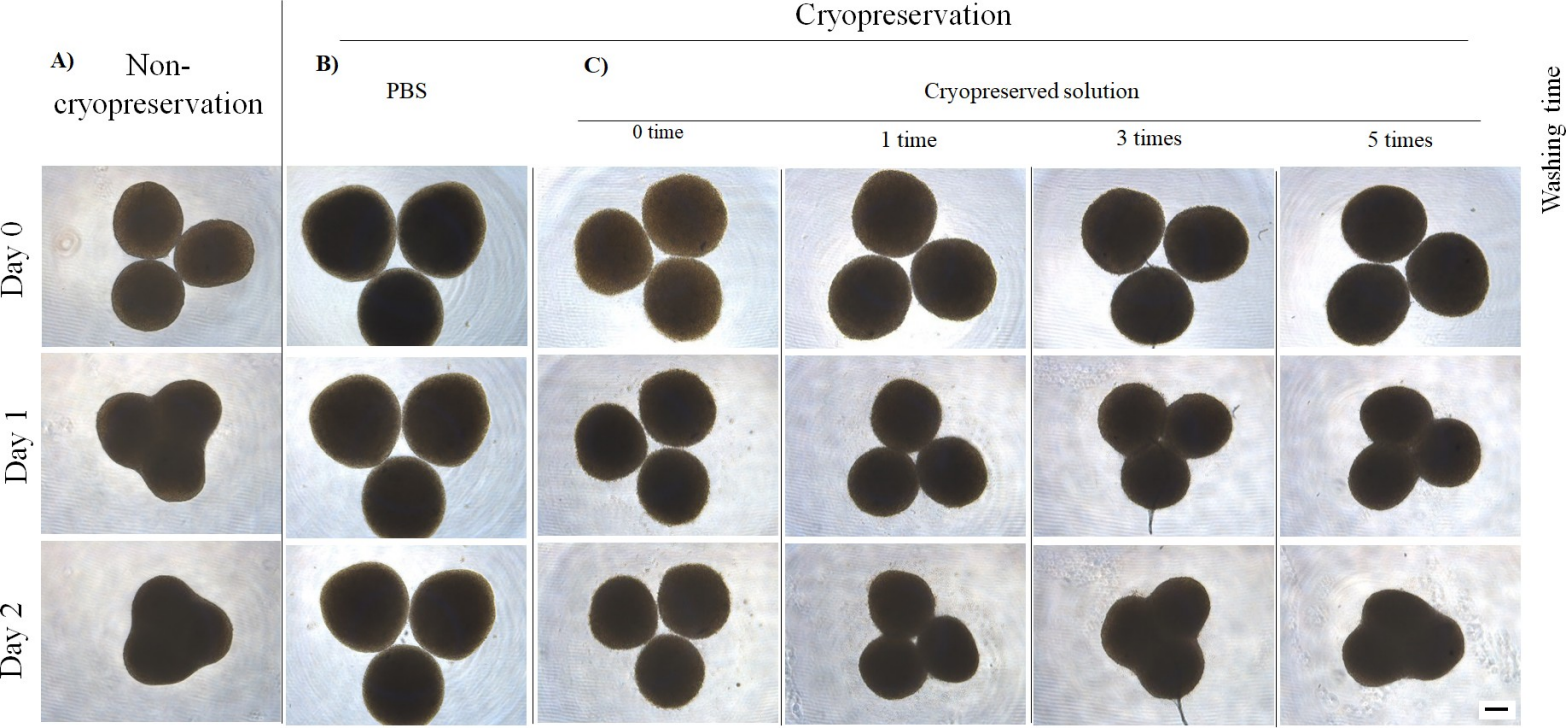
Time-lapse photograph of cryopreserved spheroids after thawing. In the positive control, non-cryopreserved spheroids were fused with each other (A). For the negative control, spheroids were cryopreserved in PBS (B). Cryopreserved spheroids in cryopreservation solution were washed 0, 1, 3, and 5 times after thawing, and fusion activities of the spheroids were analyzed (C). The scale bar is 200 μm.

We evaluated spheroid fusion to verify the optimal washing times after thawing the cryopreserved spheroids. Non-cryopreserved spheroids began to fuse on day 1 (Fig 3A), whereas spheroids cryopreserved in PBS did not fuse (Fig 3B). Among spheroids preserved in the cryopreservation solution, non-washed spheroids did not fuse, and in those washed once, fusion began on day 2; meanwhile, spheroids washed three or five times began to fuse on day 1 (Fig 3C). We confirmed the reproducibility of spheroids fusion results by repeating the experiments five times (data not shown).

**Fig 4:**
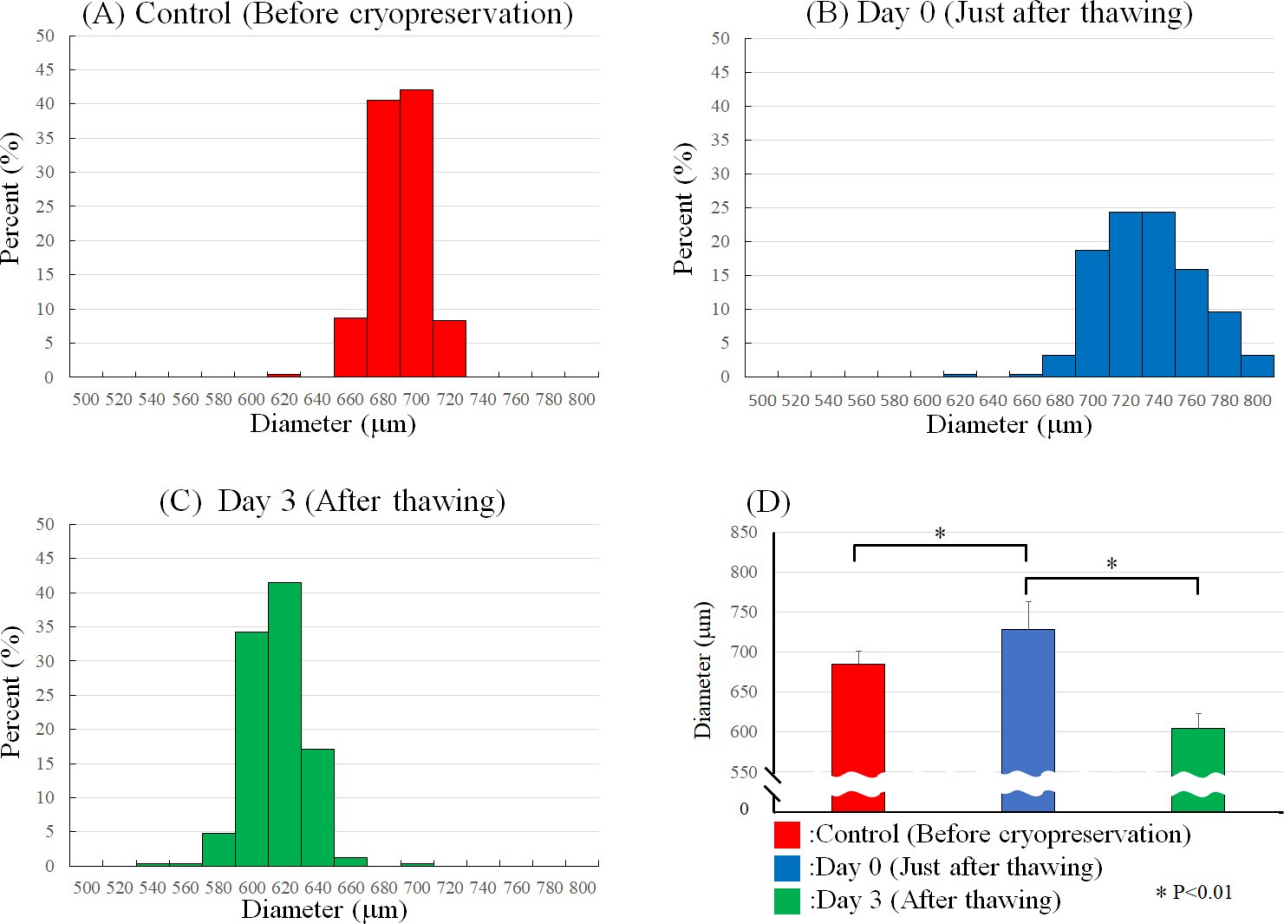
Size of cryopreserved spheroids after thawing. Spheroid sizes before cryopreservation (A), just after thawing (B), and 3 days after thawing (C) were measured with a measuring system on the bio-3D printer. The spheroid diameter comparison among spheroids before cryopreservation, just after thawing and after thawing (D). N = 239 for each group. *P<0.01.

**Fig 5:**
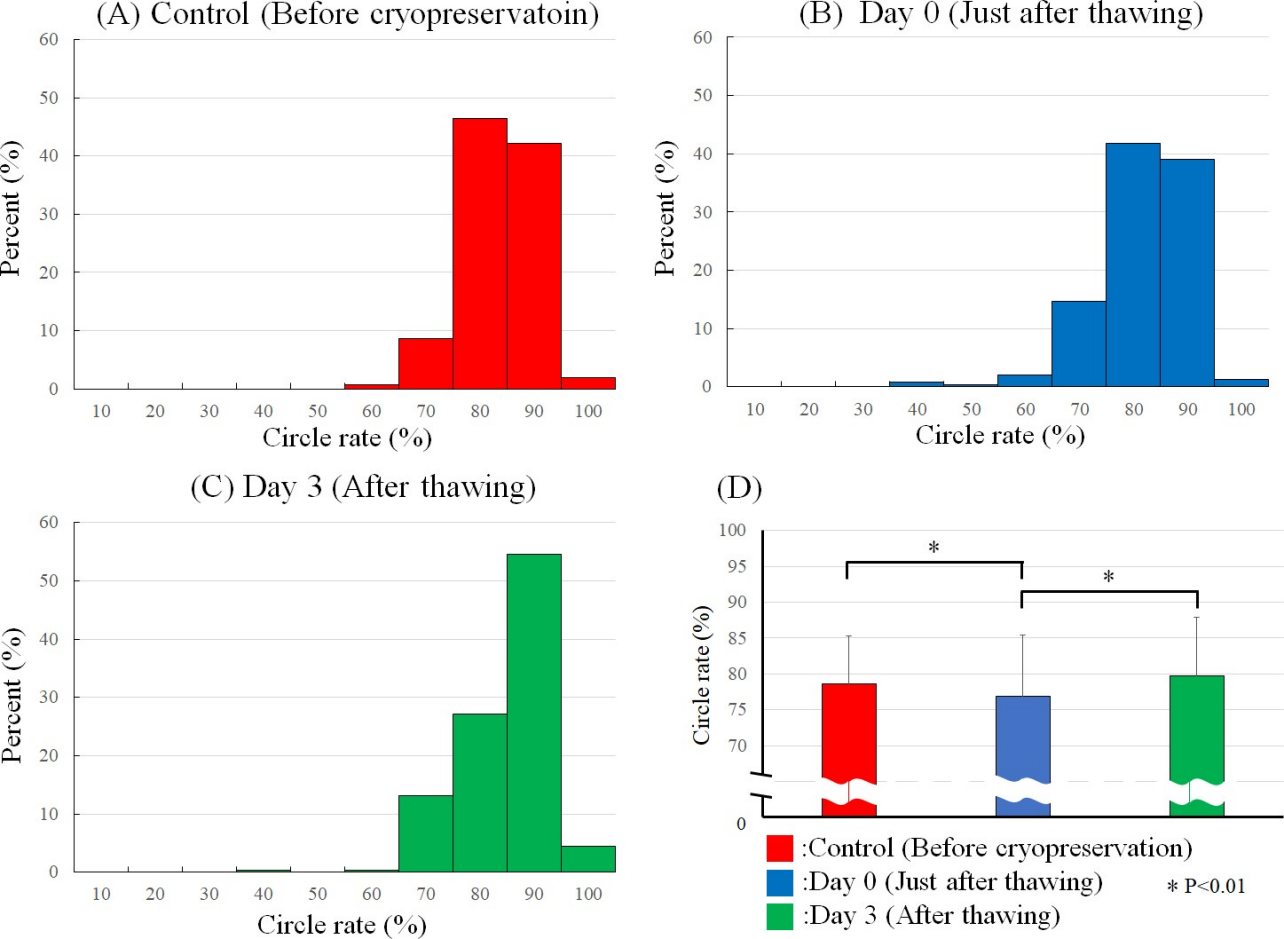
Roundness of cryopreserved spheroids after thawing. Spheroid roundness was measured before cryopreservation (A), just after thawing (B), and 3 days after thawing (C). The spheroid roundness comparison among spheroids before cryopreservation, just after thawing and after thawing (D). N = 239 for each group. *P<0.01.

In addition, the diameter and roundness of the spheroids preserved in the cryopreservation solution were measured before cryopreservation, on days 0 and 3 after thawing. The cryopreserved spheroids were thawed and observed after washing five times. The diameters of the cryopreserved spheroids increased just after thawing compared to those before cryopreservation (Fig 4A and 4B). Most spheroids shrank and became stable 3 days later (Fig 4C). The roundness of the cryopreserved spheroids was nearly stable just after thawing compared to that before cryopreservation (Fig 5A and 5B). The roundness of nearly all spheroids increased 3 days later (Fig 5C).

### 3.2. Histological analysis and cell viability of cryopreserved spheroids and tubular constructs

**Fig 6:**
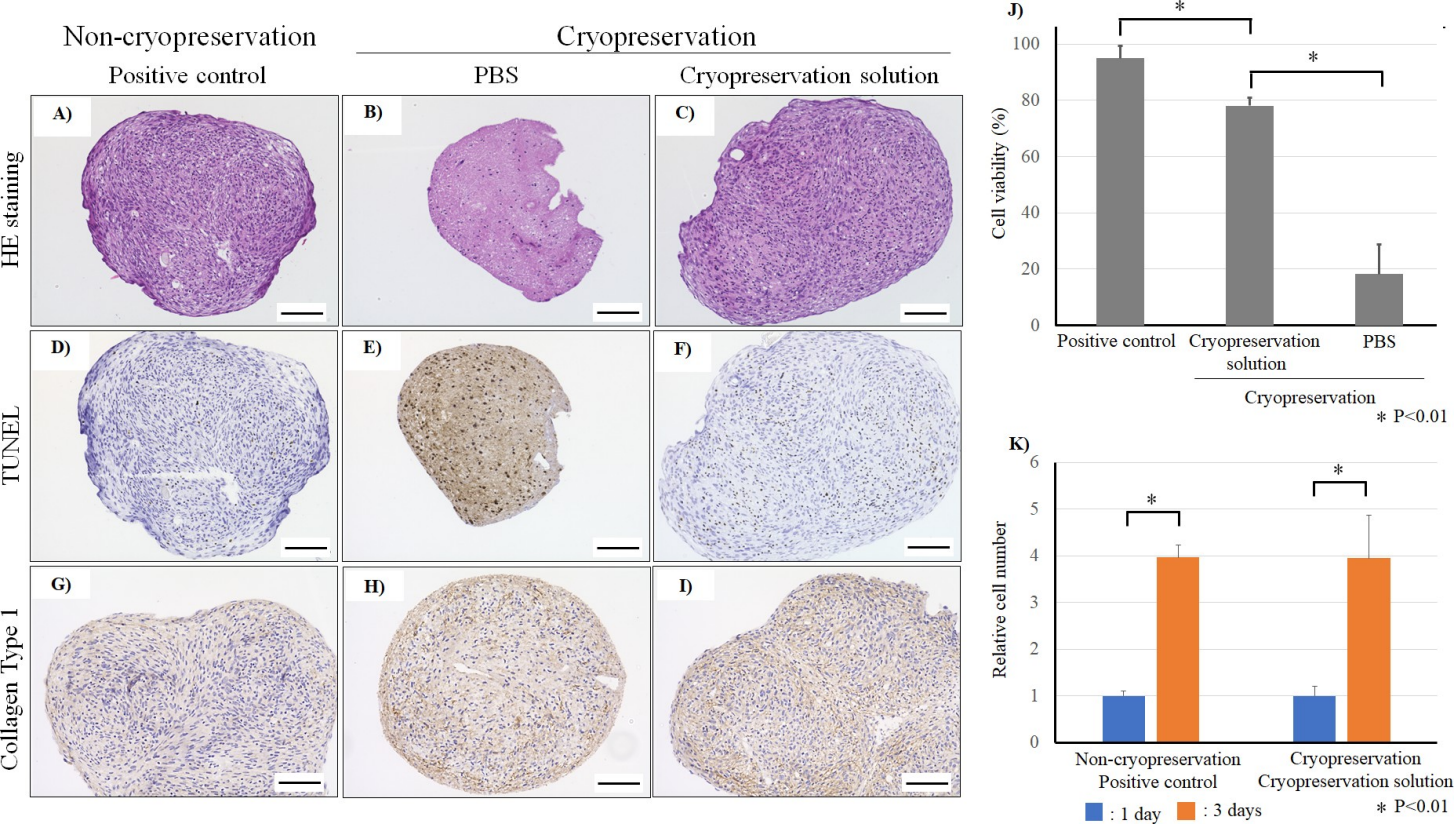
Histological analysis of spheroids. Non-cryopreserved spheroids (A), cryopreserved spheroids using PBS solution (B), and cryopreserved spheroids using StemSure® solution (C) were analyzed by hematoxylin and eosin staining. Non-cryopreserved spheroids (D), cryopreserved spheroids using PBS solution (E), and cryopreserved spheroids using StemSure® solution (F) were analyzed by TUNEL staining to detect apoptotic cells in the spheroids. Non-cryopreserved spheroids (G), cryopreserved spheroids using PBS solution (H), and cryopreserved spheroids using StemSure® solution (I) stained for collagen type Ⅰ. The scale bar is 100 μm. The number of dead cells was calculated from TUNEL staining images (K). N = 5 for each group, *P<0.01 The cell proliferation comparison between non-cryopreserved spheroids and cryopreserved spheroids (L). N = 3 for each group, *P<0.01.

**Fig 7:**
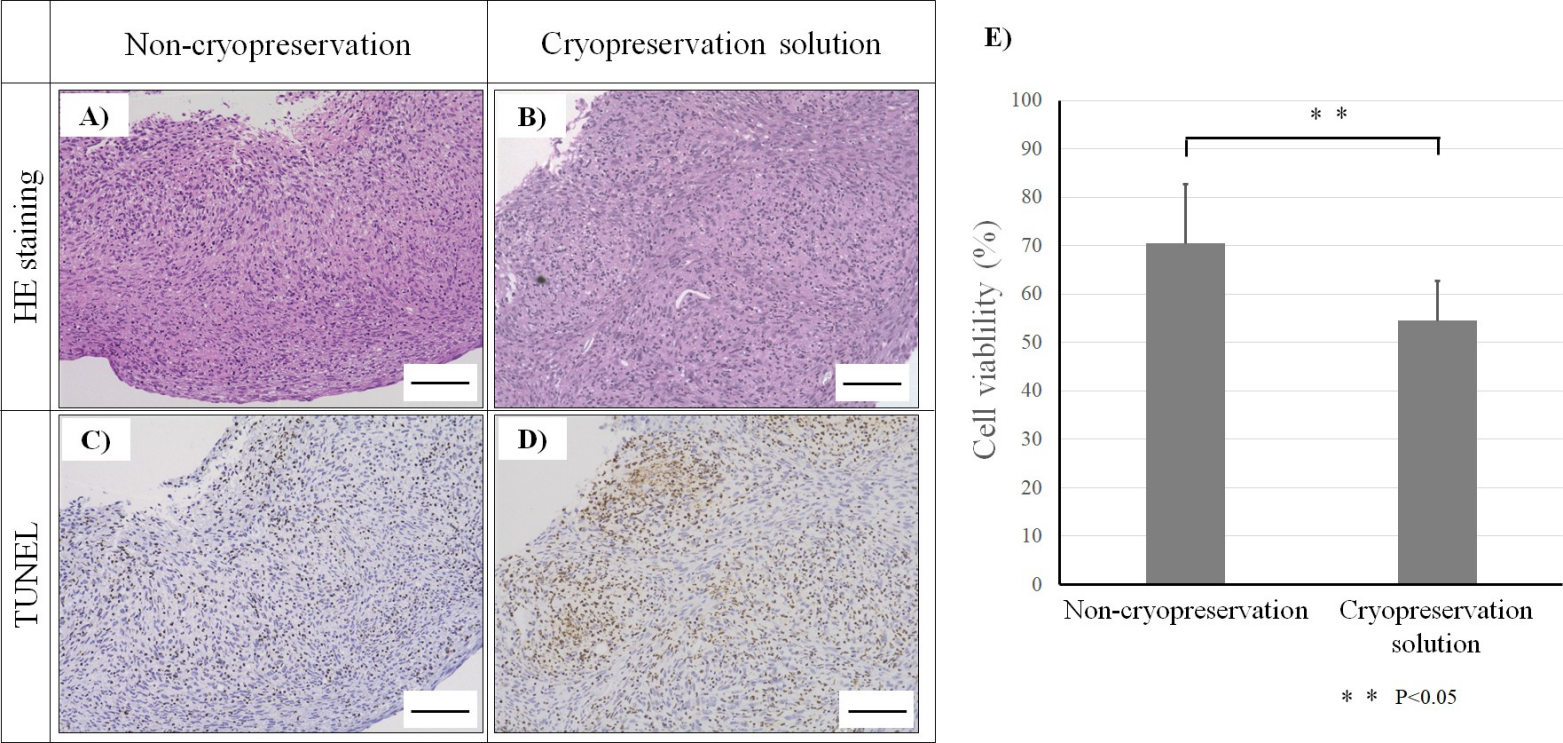
Histological analysis of constructs. Tubular constructs using non-cryopreserved spheroids (A) or cryopreserved spheroids (B) were analyzed by hematoxylin and eosin staining. These samples were analyzed by TUNEL staining to detect apoptotic cells in the construct (C and D). The scale bar is 100 μm. Cell viabilities in these constructs were measured by counting the number of dead cells (E). N = 5 for each group, **P<0.05.

Histological analysis revealed spheroid fusion and viable cells in the cryopreserved and non-cryopreserved spheroids (Fig 6). According to TUNEL staining, cell viability in the non-cryopreserved spheroids was 95% (Fig 6D), while that in cryopreserved spheroids was 78% (Fig 6F). In contrast, nearly all cells in spheroids preserved in PBS showed very low viability (Fig 6G). In addition, collagen type Ⅰ expression in non-cryopreserved and cryopreserved spheroids was observed (Fig 6G–6I). The cell proliferation capacities of non-cryopreserved and cryopreserved spheroids were measured. Cell growth rate of cryopreserved spheroid was similar to that of non-cryopreserved spheroids (Fig 6K). These data indicate that the cell viability in cryopreserved spheroids decreased, but the collagen type Ⅰ expression and cell proliferation did not change. Tubular constructs of cryopreserved or non-cryopreserved spheroids were examined by histological analysis and TUNEL staining (Fig 7). The cell density and arrangement in the tubular construct using cryopreserved spheroids were similar to those in the construct using non-cryopreserved spheroids (Fig 7A and 7B). The construct section of non-cryopreserved spheroids showed very low levels of TUNEL staining, while several cells were stained in the construct of cryopreserved spheroids (Fig 7C and 7D). Using image analysis software, we found that the number of TUNEL-positive cells in the construct using cryopreserved spheroids was greater than that of non-cryopreserved spheroids (Fig 7E).

### 3.3. Construct tensile strength and deposition of extracellular matrix

**Fig 8:**
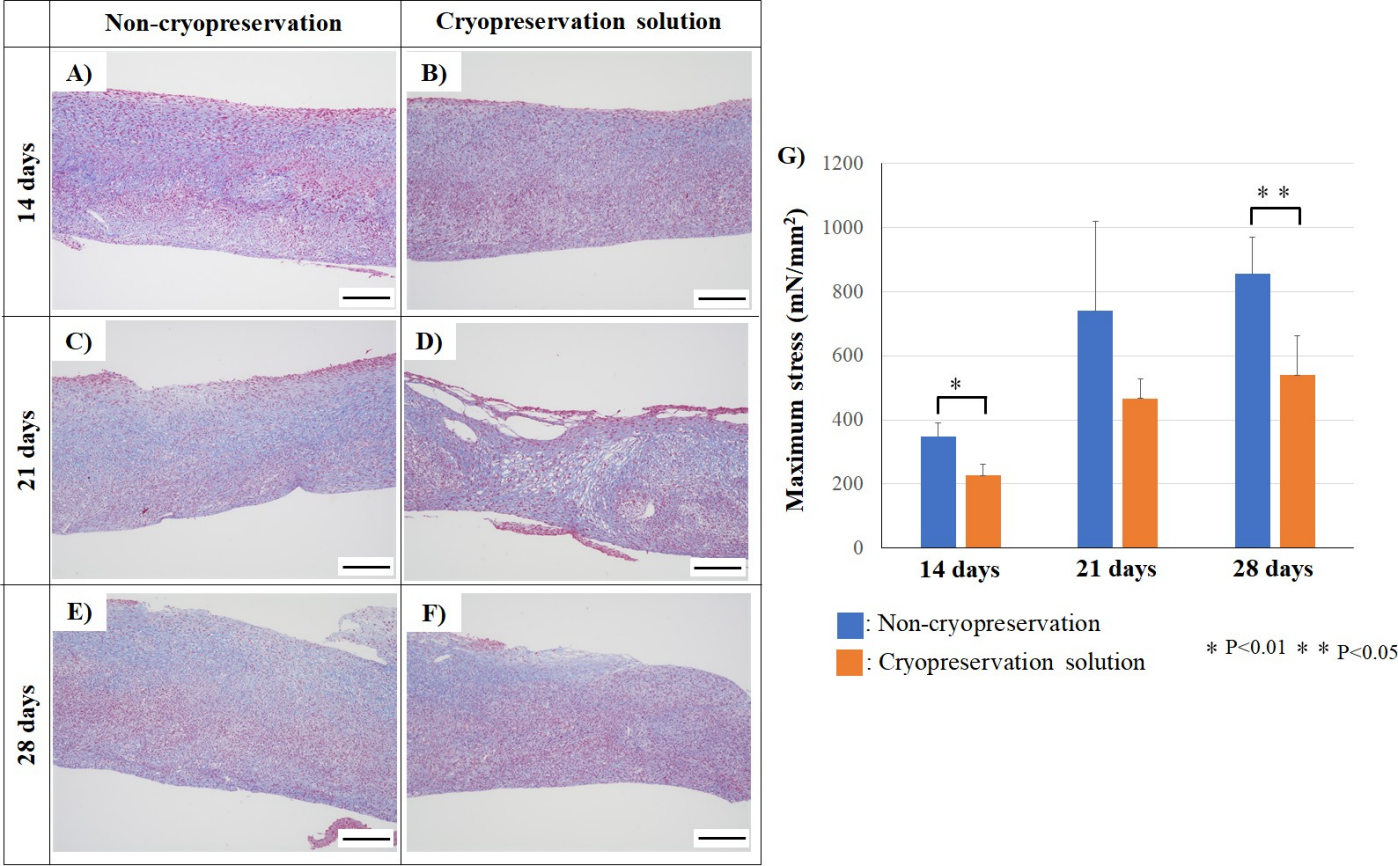
Extracellular matrix deposition and tensile strength of constructs. Constructs using non-cryopreserved spheroids or cryopreserved spheroids were analyzed by Masson’s trichrome staining at 14 days (A or B), 21 days (C or D), and 28 days (E or F). The scale bar is 200 μm. The tensile strength of the constructs was measured and analyzed (G). N = 3 for each group, *P<0.01, **P<0.05.

Masson’s trichrome staining revealed the expression of extracellular matrix (ECM) in tubular constructs. The ECM expression of constructs obtained from non-cryopreserved spheroid or cryopreserved spheroids were confirmed after days 14, 21, and 28 (Fig 8A–8F).

The tensile strength of tubular constructs using cryopreserved spheroids was evaluated (Fig 8G), which showed that the tensile strength of tubular constructs using cryopreserved spheroids was 226.9 ± 33.6 mN/mm^2^ at 14 days of culture. The tensile strength gradually increased to 467.6 ± 59.6 mN/mm^2^ and 541.1± 122.5 mN/mm^2^ after days 21 and 28, respectively. However, the tensile strength of tubular constructs using cryopreserved spheroids was lower than that of the constructs by non-cryopreserved spheroids.

## 4. Discussion

Cryopreserved spheroids should maintain the same characteristics and quality as non-cryopreserved spheroids. We focused on three basic theories to fabricate scaffold-free tissue constructs with a bio-3D printer: (1) cryopreserved spheroids can fuse; (2) the cell viability, size, and roundness of cryopreserved spheroids can be maintained; and (3) the cell viability of scaffold-free constructs using cryopreserved spheroids can be maintained.

In this study, we cryopreserved fibroblast-based spheroids using an optimized cryopreservation method and solution. We used the slow-freezing method to cryopreserve spheroids consisting of somatic cells, as this method is suitable for somatic cell-based spheroids. It is difficult to form spheroids using the vitrification method because the cooling process of the cells depends on the cooling and warming rates, sample viscosity, sample volume, and operation by researchers. A balance between these factors must be maintained to vitrify the cells [9]. We used StemSure^®^ freezing medium to cryopreserve fibroblast-based spheroids. The medium did not contain serum but instead had 10% Me_2_SO, and it was thus appropriate for preserving various types of cells, including somatic cells and stem cells.

We previously prepared scaffold-free constructs by using a bio-3D printer [17,19,20], mainly using fibroblasts. In this study, we cryopreserved fibroblast-based spheroids, but it was necessary to confirm whether the cryopreservation of other cell type-based spheroids yielded the same results.

First, we confirmed that the cryopreserved spheroids were fused. When non-cryopreserved spheroids were collected and cultured, the spheroids fused on day 1. The fusion activity of spheroids is among the most important characteristics for fabricating tissue constructs with a bio-3D printer. We found that cryopreserved spheroids can fuse using the cryopreservation method developed by us.

The four major steps in cryopreservation by slow-freezing were as follows: (1) cryopreservation solution was mixed with cells or spheroids, (2) cells or spheroids were mixed with cryopreservation solution at a low temperature (−80°C or −196°C) and stored, (3) the stored cells or spheroids were thawed, and (4) cryopreservation solution was removed from the cells or spheroids after thawing [21]. In general, the cryopreserved vial is melted by warming on a water bus. In this study, the frozen vials were thawed by adding a warmed culture medium. This is because this method reduces the time for immersing spheroids in a high-concentration cryopreservation solution. We expected to minimize the effects of cell viability in spheroids by using a warmed medium. We focused on the method of washing the spheroids after thawing in the fourth step. Although spheroids washed, three or five times began to fuse in 24 h, those washed only once began to fuse after 24 h. Furthermore, non-washed spheroids did not fuse. In general, the cryopreservation solution contains Me_2_SO, which can protect cells from freezing damage but cause cytotoxicity. With fewer washes, the cryopreservation solution remained inside the spheroids and exhibited cytotoxic effects. The StemSure^®^ freezing medium exhibits cytotoxic effects on cells in cryopreserved spheroids because this medium contains 10% Me_2_SO. If the concentration of Me_2_SO in StemSure^®^ freezing medium decreases from 10% to 5%, the viability of the cells in the cryopreserved spheroids can be maintained at a high level, and the number of washes may also be reduced.

In general, the cryopreservation solution can be removed from a single-cell suspension solution by using a centrifuge. However, the cryopreservation solution remaining inside the spheroids may affect cell viability and spheroid fusion. Maehara et al. reported a vitrification method for chondrocyte cell sheets [22]. The cell sheet was washed several times in equilibration solution and washing solution to help diffuse the cryopreservation solution inside the cell sheets after thawing to remove the cryopreservation solution and maintain osmotic pressure. Therefore, the washing process is among the crucial factors in the cryopreservation of spheroids. The diameter of the cryopreserved spheroids was not uniform and was larger than that of spheroids before being cryopreserved. The cryopreserved spheroids were larger compared to before cryopreservation because of ice crystal formation in the cytoplasmic matrix.

In this study, the cell viability of non-cryopreserved spheroids was approximately 95%, while that of cryopreserved spheroids was approximately 78%. However, the cell viability of cryopreserved and non-cryopreserved spheroids should be the same. These results indicate that the cell viability of spheroids was reduced by approximately 17% after cryopreservation. Cryopreservation of suspended cells reduced their viability by approximately 13% compared to that before cryopreservation. In future studies, the cell viability of cryopreserved spheroids should be improved to reach the same level as that of suspended cells.

Finally, we fabricated tubular constructs using cryopreserved spheroids. Nevertheless, another method for fabricating a scaffold-free tubular construct was presented by Strobel et al., who reportedly fabricated tissue rings by seeding living cells in agarose mold, in turn, generating tubular constructs by connecting several tissue rings [23]. Although this method proved useful for fabricating tubular-shape-construct, it is still difficult to use for fabricating complex-shaped, large, and thick constructs. Conversely, our bio-3D printer-assisted method can fabricate complex-shaped constructs by loading spheroids onto needle array, in a fully-customizable fashion, according to the desired 3D design. Although in this study we fabricated simpler constructs, we consider that the present method, which was developed to fabricate constructs using a bio-3D printer based on cryopreserving spheroids, has the potential to fabricate complex-shaped construct; this will be the goal of future studies. At first, spheroids with an optimal size and roundness should be prepared to form a construct with a bio-3D printer because the pick-up nozzle for spheroids in the printer cannot function properly when spheroids are too small [13]. However, the diameter of the cryopreserved spheroids increased just after thawing compared to before cryopreservation in this study. Nearly all cryopreserved spheroids shrank after culture for 3 days. We placed the cryopreserved spheroids on the needle array and confirmed that spheroids fused after cultivation. These cryopreserved spheroids in fabricated tubular constructs were confirmed to fuse after 7 days. Thus, the cryopreservation of spheroids has minimal effects on spheroid fusion. Cell viability in constructs using non-cryopreserved spheroids was approximately 70±12%. In contrast, the viabilities of cells in constructs prepared from cryopreserved spheroids were 54±8%. The viabilities of cells in constructs using non-cryopreserved spheroids were 25% lower than those of cells in non-cryopreserved spheroids. Similarly, the viabilities of cells in constructs using cryopreserved spheroids were 23.5% lower than those of cells in cryopreserved spheroids. Lin et al. reported that spheroids with a diameter greater than 500 μm contain a necrotic core in the inner region of the spheroids [24]. Thus, the decreased cell viabilities in the constructs using cryopreserved spheroids were likely related to both spheroid cryopreservation and construct thickness.

Although cell viability in the constructs using cryopreserved spheroids decreased, whether these viabilities are important for the clinical application depends on each tissue. For example, the heart and liver must maintain cell viability and high density in constructs because the cells play functional roles. It is also vital for blood vessels to have sufficient mechanical strength for clinical applications [25]. Our research group has reported the regeneration of several target tissues (blood vessel, diaphragm, and conduits for nerve regeneration) using fibroblast-based constructs. We compared the ECM expression and the mechanical strength of the constructs prepared using cryopreserved spheroids to those made using non-cryopreserved spheroids for tissue regeneration. Although the ECM expression of the constructs obtained from non-cryopreserved spheroids and cryopreserved spheroids could be confirmed, the tensile strength of the constructs using cryopreserved spheroids was lower than that of constructs obtained from non-cryopreserved spheroids. The tensile strength of the constructs using cryopreserved spheroids might be lower than those of non-cryopreserved spheroids because of lower cell viability of cryopreserved spheroids. In general, the tensile strength of the tissues is related to the amount of ECM in the tissues. Since the tensile strength of the constructs increased with the culture time, we argue that the ECM expression in the constructs increased in a time-dependent manner. The mechanical strength of constructs using cryopreserved spheroids might be improved in the future with more advanced cryopreservation methods for spheroids and long-term cultivation of the constructs. The constructs using cryopreserved spheroids must have the same mechanical strength as those prepared using non-cryopreserved spheroids when used in clinical applications.

Our method developed for cryopreserving spheroids can be clinically applied in cell-based therapies. Furthermore, spheroids can be cryopreserved, and constructs can be fabricated by improving this technology.

## Supporting information

S1 Data Cryopreservation method for spheroids and fabrication of scaffold-free tubular constructs minimal data set. (PDF)
