# Correction: Cryopreservation method for spheroids and fabrication of scaffold-free tubular constructs

**DOI:** 10.1371/journal.pone.0243249

**Published:** 2020-11-30

**Authors:** Kenichi Arai, Daiki Murata, Shoko Takao, Ana Raquel Verissiomo, Koichi Nakayama

There is an error in the Competing Interests statement. The correct Competing Interests statement is as follows: Co-author K. Nakayama is a co-founder and shareholder of Cyfuse Biomedical KK. and an inventor/ developer designated on the patent for the Bio-3D printer. Patent title: Method for Production of Three-Dimensional Structure of Cells; patent number: JP4517125. Patent title: Cell Structure Production Device; patent number: JP5896104. The other authors have declared that no competing interests exist. This does not alter the authors' adherence to PLOS ONE policies on sharing data and materials.
