# Supplementary material for: Cryopreservation method for spheroids and fabrication of scaffold-free tubular constructs
Source: PLoS One. 2020 Apr 2;15(4):e0230428. doi: 10.1371/journal.pone.0230428 (PMC7117714; doi:10.1371/journal.pone.0230428)
Supplement: S1 Data — (PDF) [file pone.0230428.s001.pdf]

Cryopreservation method for spheroids and fabrication of scaffold-free tubular constructs
Minimal data set

Fig.#	Mean	S.D	Statistical method used	P value	# samples
**Fig.4D**			Paired two-tailed T test	*P<0.01	
Control(Before cryopreservation)	684.7273	16.64505			239
Day0(Just after thawing)	727.6347	35.72251			239
Day3(After thawing)	605.0131	17.69579			239
**Fig.5D**			Paired two-tailed T test	*P<0.01	
Control(Before cryopreservation)	78.59323	6.718732			239
Day0(Just after thawing)	76.82762	8.577055			239
Day3(After thawing)	79.76534	8.143372			239
**Fig.6J**			Paired two-tailed T test	*P<0.01	
Positive control	95	4.4			5
Cryopreservation solution	78	2.868798			5
PBS	18.16667	10.71557			5
**Fig.6K**			Paired two-tailed T test	*P<0.01	
Non-Cryopreservation 1day	0.999883	0.109813			3
Non-Cryopreservation 3days	3.964778	0.267632			3
Cryopreservation 1day	1	0.211234			3
Cryopreservation 3days	3.946246	0.916455			3
**Fig7.E**			Paired two-tailed T test	*P<0.05	
Non-Cryopreservation	70.46667	12.20669			5
Cryopreservation solution	54.53333	8.24702			5

**FIg8.G**			Paired two-tailed T test	*P<0.01, **P<0.05	
Non-Cryopreservation 14 days	349.1	42.86759			3
Cryopreservation solution 14days	226.8667	33.5673			3
Non-Cryopreservation 21days	740	280.5828			3
Cryopreservation solution 21days	467.5667	59.60506			3
Non-Cryopreservation 28days	854.9	114.3155			3
Cryopreservation solution 28days	541.1333	122.4915			3
